# Correlation Between Skin-to-Subarachnoid Space Depth in the Lumbar Region Measured by Ultrasound and Needle Insertion Depth: A Prospective Observational Study

**DOI:** 10.7759/cureus.91826

**Published:** 2025-09-08

**Authors:** Thameem Ahamed M, Udhaya Sankar Sethuraman, Thirumurugan Arikrishnan, Suneeth P Lazarus

**Affiliations:** 1 Anesthesiology and Critical Care, Sri Manakula Vinayagar Medical College and Hospital, Puducherry, IND

**Keywords:** lumbar puncture, needle insertion, spinal anaesthesia, stocker's formula, subarachnoid space, ultrasonography

## Abstract

Background and aim

Lumbar puncture and spinal anesthesia are critical procedures in medical practice, requiring precise needle placement to access the subarachnoid space. This study aimed to assess the correlation between ultrasound (USG) measurements of skin-to-subarachnoid space depth (SSD) and actual needle insertion depth.

Methods

The study received ethical approval from the institutional review board. Informed consent was obtained from all participants. This prospective observational study was conducted in a tertiary care hospital, involving 100 adult patients undergoing spinal anesthesia. USG measurements of SSD were taken, and actual needle insertion depths were recorded. Statistical analysis included Pearson correlation coefficients, independent t-tests, and one-way ANOVA, with a significance level set at p < 0.05.

Results

The mean SSD measured by USG was 4.51 cm (SD = 0.62), while the actual needle insertion depth was 4.57 cm (SD = 0.62). A strong positive correlation was found between USG measurements and actual needle depth (r = 0.94, p < 0.001). Stocker’s formula showed a correlation coefficient of r = 0.86 (p < 0.001).

Conclusions

USG guidance significantly improves the accuracy of SSD measurements compared to traditional methods, including Stocker’s formula, thereby enhancing the safety and efficacy of lumbar puncture procedures.

## Introduction

Spinal anesthesia is a commonly performed regional anesthetic technique in surgeries involving the lower abdomen and lower limbs due to its rapid onset, dense neural blockade, and cost-effectiveness [1]. Precise identification of the subarachnoid space is critical for the success of this technique. Traditionally, surface anatomical landmarks have been used to guide needle insertion, but these may be unreliable, especially in patients with obesity, spinal deformities, or poorly palpable spinous processes.

To overcome such limitations, ultrasound (USG) has emerged as a valuable preprocedural tool for identifying spinal structures [2]. It provides real-time visualization of the vertebral anatomy, enabling accurate measurement of the skin-to-subarachnoid space depth (SSD). USG guidance improves the first-pass success rate, reduces needle attempts and redirections, and increases patient comfort [3]. Meanwhile, Stocker’s formula (SSD (in mm) = 0.5 × weight [kg] + 18) [4] offers a simplified method of estimating SSD based on patient body weight, which can be useful in resource-limited settings. Several formulas, including Stocker’s, have been evaluated for their predictive value, with varying degrees of correlation to actual measured depths [5].

We hypothesized that USG-guided measurement of SSD would demonstrate superior correlation with actual needle insertion depth compared to Stocker’s formula, based on existing literature. Hence, the current study evaluates the correlation between USG-measured SSD and actual needle insertion depth during spinal anesthesia, while also comparing predictions from Stocker’s formula. This study aims to validate USG’s accuracy in our local population and compare it with Stocker’s formula, thereby contributing to the external validation of USG-guided depth estimation in spinal anesthesia, particularly in South Indian patients.

## Materials and methods

This prospective observational study was conducted in the Department of Anesthesiology at Sri Manakula Vinayagar Medical College and Hospital, which serves a large population in the outskirts of Pondicherry. It was carried out over 18 months, from May 2023 to December 2024, after approval from the Institutional Ethics Committee (EC/43/2022) and CTRI (CTRI/2023/05/052966). Written informed consent was obtained from all participants in their local language, ensuring autonomy, confidentiality, and nonmaleficence. The study aimed to compare USG-predicted depth to the subarachnoid space with the actual needle insertion depth during spinal anesthesia, thereby evaluating USG as a predictive tool in anesthetic practice.

Inclusion criteria

Patients were eligible if they voluntarily provided informed consent, were aged 18-60 years, were of any gender, had an American Society of Anaesthesiologists (ASA) Physical Status classification of I or II (indicating a healthy individual or one with mild systemic disease), and were scheduled for elective surgery requiring spinal anesthesia.

Exclusion criteria

Patients were excluded from the study if they were unwilling to provide informed consent for spinal anesthesia or had any absolute contraindications to the procedure. These contraindications included local infection at the injection site, bleeding disorders, increased intracranial pressure, and fixed cardiac output states. Additionally, patients with spinal abnormalities that would complicate the procedure, those requiring a paramedian approach, and those undergoing emergency surgeries were also excluded.

**Figure 1:**
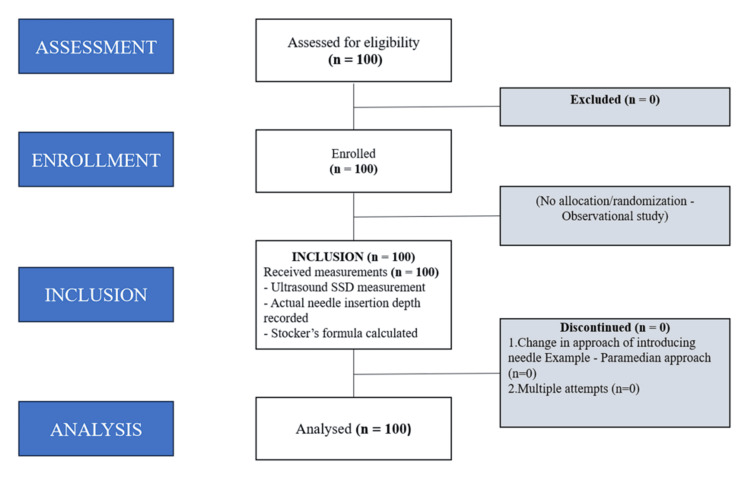
STROBE flow diagram SSD, skin-to-subarachnoid space depth; STROBE, STrengthening the Reporting of OBservational studies in Epidemiology

A total of 100 patients were analyzed by convenience sampling. Although randomization was not feasible in this observational design, selection bias was minimized by consecutively including all eligible patients (Figure 1).

Sample size was based on Nair et al. [6] using OpenEpi, Version 3, with sample size calculation for the two independent means method, thereby considering the mean and standard deviation of ultrasonographic and actual insertion depths (4.81 ± 0.6 vs. 4.47 ± 0.6). With 80% power and 95% CI, the calculated size was 98, rounded to 100. The primary outcome was actual insertion depth compared with preoperative USG-measured SSD.

Preoperative and patient preparation

Patients were kept nil per os for eight hours prior to the procedure. Premedication included oral alprazolam 0.5 mg and oral pantoprazole 40 mg. Informed consent was obtained from each participant, and demographic, comorbidity, and baseline vital signs were recorded. On the day of surgery, patients were moved to a holding area for one hour before being transferred to the OR. In the OR, standard monitoring (noninvasive blood pressure, pulse oximetry, and ECG) was initiated, and an IV line was secured to administer fluids.

Ultrasonographic assessment

**Figure 2:**
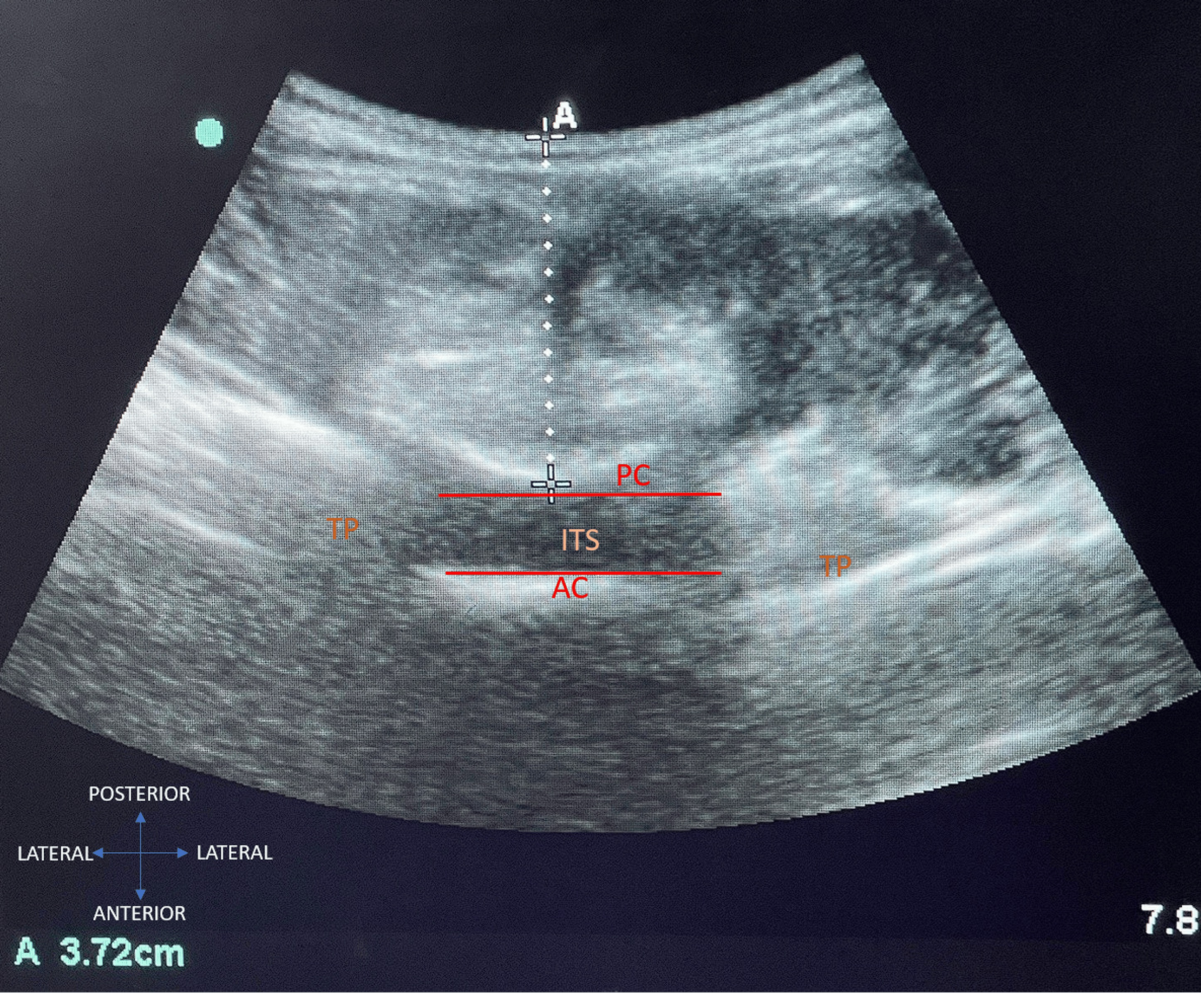
USG measurement with built-in caliper AC, anterior complex; ITS, intrathecal space; PC, posterior complex; TP, transverse process; USG, ultrasound

The patient was positioned seated on the operating table, and ultrasonographic measurements were performed using a SONOSITE USG system with a 2-5 MHz curvilinear array transducer. The transducer was placed in the median sagittal plane over the sacrum to visualize the hyperechoic line corresponding to the sacral image. The probe was moved cephalad to identify the interspinous spaces until the L3-L4 interspace was located, followed by a transverse scan to confirm the absence of an acoustic shadow and visualize the ligamentum flavum-dura mater complex (posterior complex) (Figure 2) and vertebral body. The outlines of the target structures were marked with a sterile dermographic pen, and the optimal sonogram was captured and recorded. The built-in caliper was used to measure the distance from the skin to the anterior part of the ligamentum flavum-dura mater complex, marking the lower limit before the intrathecal space. For the study, the SSD was calculated using Stocker’s formula: SSD (mm) = 0.5 × weight (kg) + 18 and recorded.

Spinal anesthesia procedure

Following the USG assessment, the patient was prepared for a lumbar puncture in a seated position with knees flexed. The lumbosacral region was prepped with povidone-iodine and spirit, and the L3-L4 interspace was manually palpated. The area was infiltrated with 2% lignocaine before a 23G Quincke’s needle was advanced until free flow of CSF confirmed entry into the subarachnoid space. An appropriate dose and volume of local anesthetic was administered intrathecally at a rate of 0.2 ml/second, after which the needle was withdrawn. The depth of the needle at which it was inserted was marked by a sterile marker and measured using Vernier calipers.

Postoperative monitoring and management

Following the procedure, patients were placed in a supine position with continuous monitoring of vital signs. Sensory and motor blockade levels were assessed every two minutes using the pin-prick test and the modified Bromage scale, respectively, until maximum blockade was achieved. Hemodynamic parameters were recorded at predetermined intervals. Bradycardia (heart rate < 50 bpm) was treated with 0.6 mg IV atropine, and hypotension (mean arterial pressure decrease > 20%) was managed with 6 mg IV ephedrine. Perioperative analgesia was provided with 15 mg/kg IV paracetamol, and any adverse events were documented and reported to the ethics committee within 24 hours.

USG measurements and lumbar punctures were performed by a single experienced anesthesiologist to ensure consistency and reduce interobserver variability. Data were recorded in predesigned proformas, entered in MS Excel, and analyzed using IBM SPSS Statistics for Windows, Version 20.0 (Released 2011; IBM Corp., Armonk, NY, USA). Normality was tested with the Shapiro-Wilk test. Student’s t-test or Mann-Whitney U-test, Pearson or Spearman correlation, and chi-square test were applied as appropriate. P < 0.05 was considered statistically significant. Agreement between methods was evaluated using Bland-Altman analysis, including calculation of bias and 95% limits of agreement. Subgroup analyses for gender differences utilized independent t-tests, while BMI-stratified comparisons employed one-way ANOVA with Bonferroni post hoc correction for multiple comparisons.

## Results

Study population and demographic characteristics

A total of 100 participants were enrolled in this prospective observational study examining the correlation between USG-guided measurements of SSD, actual depth (AD) of needle insertion during spinal anesthesia, and predictions made using Stocker’s formula.

The study population demonstrated a mean age of 40.4 ± 11.09 years (range: 18-60 years) with a predominant male representation of 75% (n = 75). The mean (SD) weight and height were found to be 64.5 ± 13.16 kg and 161.45 ± 7.26 cm with a BMI of 24.65 ± 4.56 kg/m². According to the ASA physical status classification, 32% of patients were classified as ASA I, while 68% were ASA II, indicating a predominance of patients with mild systemic disease.

Correlation between measurement methods

The descriptive analysis revealed remarkable consistency across all three measurement methods. USG-guided measurements demonstrated a mean depth of 4.51 ± 0.62 cm (range: 3.17-6.49 cm), the actual needle insertion depth showed a mean of 4.57 ± 0.62 cm (range: 3.4-6.7 cm), and Stocker’s formula calculations yielded a mean of 4.55 ± 0.76 cm (range: 3.04-6.6 cm), and all the parameters were normally distributed (Shapiro-Wilk test, p-value >0.05).

The study demonstrates a robust agreement between the various methods used to estimate the depth of needle insertion during spinal anesthesia. The findings presented in Table 1 demonstrate a high degree of consistency among the three methods used to measure the depth of the subarachnoid space. The mean measurements were remarkably similar: 4.51 cm for USG guidance, 4.57 cm for the AD of needle insertion, and 4.55 cm for Stocker’s formula. The standard deviations were also comparable, indicating a consistent spread of data for each method. A one-way ANOVA analysis was applied following the normality testing with the Shapiro-Wilk test (>0.05). The analysis revealed no statistically significant difference in needle insertion depth between USG, the AD of insertion, and the depth calculated by Stocker’s formula (p = 0.8158). This outcome is further supported by the negligible effect size (partial η² = 0.001), which confirms that the choice of method did not have a substantial impact on the measured depth. The similarity in means and the nonsignificant p-value suggest that, in this study, the choice of method (USG, AD, or Stocker’s formula) did not substantially affect the depth of needle insertion.

**Table 1:** SSD measured with three different methods One-way ANOVA was applied. SSD, skin-to-subarachnoid space depth

Variables	Mean	SD	Minimum	Maximum	p-Value
USG measurement (cm)	4.51	0.62	3.17	6.49	0.8158
Actual needle insertion depth (cm)	4.57	0.62	3.4	6.7	
Depth calculated using Stocker’s formula (cm)	4.55	0.76	3.04	6.6	

USG measurements showed a very strong positive correlation with the actual needle insertion depth, with a Pearson correlation coefficient (r) of 0.94 and a highly significant p-value of <0.001, indicating excellent reliability of USG in predicting the skin-to-subarachnoid space distance. Similarly, Stocker’s formula also exhibited a strong positive correlation with the AD, with an r value of 0.86 and a p-value of <0.001, validating its predictive value based on patient anthropometric parameters. Moreover, a strong correlation (r = 0.87, p < 0.001) was observed between the depth estimated by USG and that calculated using Stocker’s formula, suggesting that both methods are in strong agreement and can reliably estimate the depth of spinal needle insertion (Table 2).

**Table 2:** Correlational analysis between different approaches of skin-to-subarachnoid space measurement The Pearson correlation test was applied. AD, actual depth

Correlational analysis		r	p-Value
USG measurement (cm)	AD of the needle (cm)	0.94	<0.001
USG measurement (cm)	Depth calculation by Stocker’s formula (cm)	0.87	<0.001
AD of the needle (cm)	Depth calculation by Stocker’s formula (cm)	0.86	<0.001

Post Hoc Analysis

Games-Howell post hoc testing confirmed no statistically significant differences between any paired comparisons: USG vs. AD (p = 0.8080), USG vs. Stocker’s formula (p = 0.9122), and AD vs. Stocker’s formula (p = 0.9884).

Agreement Analysis

**Figure 3:**
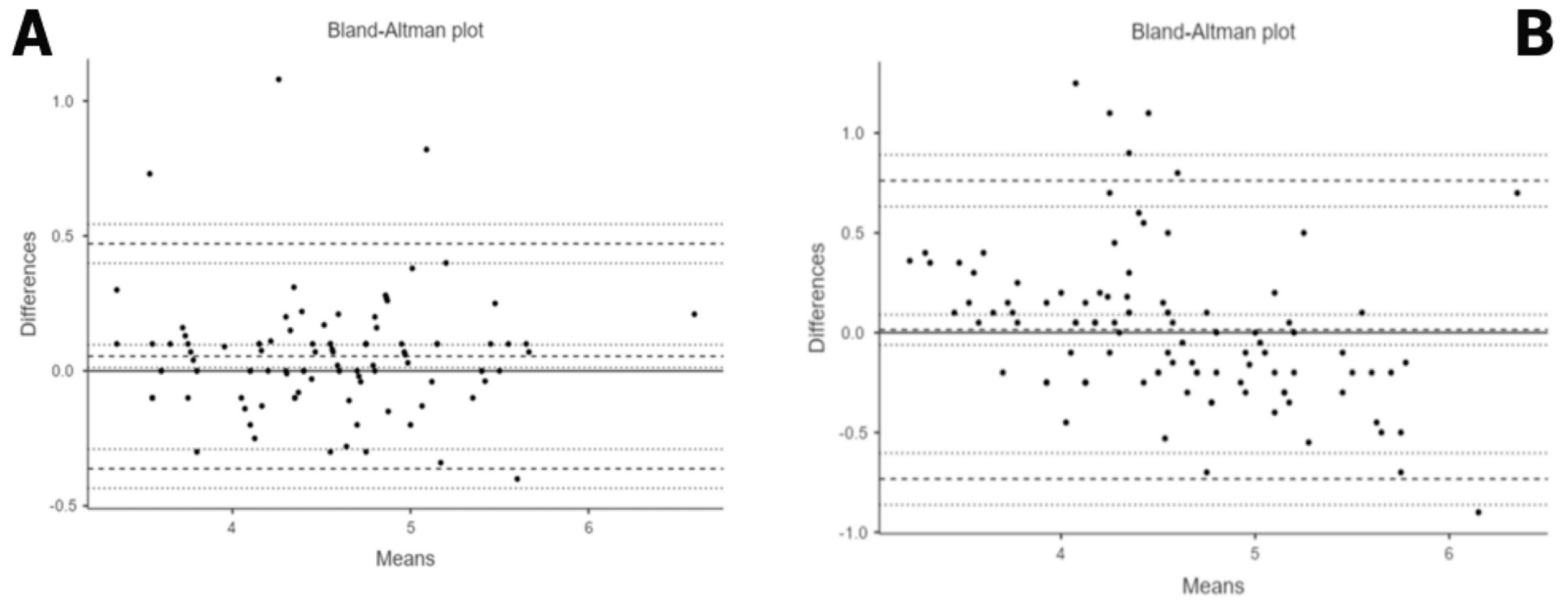
(A) Bland-Altman plot AD vs. USG-estimated depth. (B) AD vs. Stocker’s formula AD, actual depth; USG, ultrasonography

A Bland-Altman analysis was performed to evaluate the agreement between two methods for estimating spinal needle insertion depth, USG and Stocker’s formula, compared to the AD. The results (Figure 3) showed that both methods had a minimal systematic bias, suggesting that neither consistently over- nor underestimated the true depth on average. However, the USG-guided measurement demonstrated superior precision with substantially narrower limits of agreement (-0.4712 to 0.3623 cm) compared to Stocker’s formula (-0.7331 to 0.7617 cm) (Table 3). The data indicate that, while both methods are unbiased on average, USG provides a more reliable and clinically accurate depth estimation with significantly less individual variation.

**Table 3:** Bland-Altman analysis of AD of spinal needle insertion AD, actual depth; USG, ultrasound

Parameter	AD vs. USG-estimated depth		AD vs. Stocker’s formula	
	Estimate	95% CI	Estimate	95% CI
Bias (n = 100)	-0.0545	-0.097 to 0.012	0.0143	-0.061 to 0.09
Lower limit of agreement	-0.4712	-0.544 to -0.399	-0.7331	-0.863 to -0.603
Upper limit of agreement	0.3623	0.29 to 0.435	0.7617	0.762 to 0.632

Influence of patient characteristics

Gender-Related Effects

On subgroup analysis (Table 4), males demonstrated numerically greater depths across all methods; these differences did not reach statistical significance after applying Bonferroni correction for three comparisons (corrected α = 0.05/3 = 0.0167).

**Table 4:** Gender-related effects An independent t-test was applied. AD, actual depth; USG, ultrasound

Variable	Male		Female		p-Value
	Mean	SD	Mean	SD	
USG measurement (cm)	4.5826	0.5624	4.2972	0.7354	0.0453
AD of needle insertion (cm)	4.6196	0.5688	4.404	0.7391	0.1321
By Stocker’s formula (in cm)	4.6232	0.7701	4.336	0.7184	0.104

Age-Related Effects

Correlation analysis between patient age and depth measurements showed weak inverse relationships across all methods; however, after applying the Bonferroni correction for six comparisons (corrected α = 0.0083), none of the correlations remained statistically significant: age vs. USG measurements (r = -0.20, p = 0.047; not significant after correction), age vs. actual needle depth (r = -0.20, p = 0.050; not significant after correction), and age vs. Stocker’s formula (r = -0.22, p = 0.028; not significant after correction).

While there appeared to be a subtle trend toward decreased subarachnoid space depth with advancing age, this relationship was not statistically robust after correction for multiple testing.

Height Correlation Analysis

Patient height showed moderate positive correlations with all depth measurements. After applying the Bonferroni correction for multiple comparisons (corrected α = 0.0083), the statistical significance of these associations depends on the exact p-values: height vs. USG measurements (r = 0.40, p < 0.01), height vs. actual needle depth (r = 0.42, p < 0.01), and height vs. Stocker’s formula (r = 0.47, p < 0.01).

These findings suggest a direct relationship where increased patient height necessitates proportionally deeper needle insertion, though definitive significance after correction depends on whether p-values are ≤ 0.005.

BMI Stratified Analysis

A stratified analysis by BMI revealed a strong and consistent influence on all depth measurements. A one-way ANOVA demonstrated a highly significant association between BMI and depth across all three measurement methods (p < 0.001) (Table 5).

**Table 5:** BMI stratified analysis AD, actual depth; USG, ultrasound

Variable	BMI			p-Value
	Underweight	Normal	Obese	
USG measurements (in cm)	3.82 ± 0.43	4.32 ± 0.49	4.85 ± 0.59	<0.001
AD of needle insertion (in cm)	3.9 ± 0.40	4.35 ± 0.48	4.92 ± 0.59	<0.001
By Stocker’s formula (in cm)	3.74 ± 0.49	4.24 ± 0.61	5.05 ± 0.66	<0.001

Post hoc analysis with Bonferroni correction further clarified these findings. While there were no significant differences between the underweight and normal weight groups across any method (p > 0.0056), highly significant differences were maintained between the underweight and obese groups (p < 0.001) and between the normal weight and obese groups (p < 0.001) for all three measurement modalities.

## Discussion

Accurate determination of lumbar SSD is of paramount importance for optimizing the outcomes of spinal anesthesia. Precise SSD measurement contributes to a reduction in complications associated with multiple needle insertion attempts. Repeated needle penetrations increase the risk of several adverse events, including post-dural puncture headaches, traumatic taps, and lower back pain [6,7].

In this study, USG measurements, actual needle insertion depth, and Stocker’s formula consistently provided similar mean values for SSD, with no statistically significant differences. A very strong positive correlation was observed between USG measurements and actual needle depth (r = 0.94). Strong positive correlations were also found between USG and Stocker’s formula (r = 0.87), as well as between AD and Stocker’s formula (r = 0.86). All correlations were statistically significant (p < 0.001). Bland-Altman analysis indicated that while both USG and Stocker’s formula were unbiased estimators, USG guidance was more precise, as evidenced by its narrower limits of agreement. These findings confirm the high reliability and precision of USG for estimating spinal needle depth, particularly in patients with higher BMI, where accurate depth prediction is crucial.

Our findings align with and, in some cases, exceed the correlations reported in existing literature. The observed correlation of r = 0.94 between USG measurements and actual needle depth is exceptionally strong and places our study among the highest reported in systematic reviews and individual studies. For instance, a 2014 systematic review by Perlas et al. [7] reported correlations ranging from 0.83 to 0.99, with most studies exceeding 0.90 [7-9]. This consistency across a range of populations, including overweight and obstetric patients, strongly supports the excellent predictive accuracy of USG guidance.

Further, the study had revealed that the correlations of r = 0.87 (USG vs. Stocker’s) and r = 0.86 (actual vs. Stocker’s) are notably stronger than those reported in most other studies. For instance, Nair et al. [6] found a much weaker correlation of approximately 0.61. Existing evidence has consistently demonstrated correlations for Stocker’s formula in the 0.6 to 0.8 range, demonstrating that its agreement with AD is typically weaker than that of USG [6,7]. The unusually strong correlations in our study may be attributed to a particularly well-matched study population or the rigorous methodological approach employed.

The Bland-Altman analysis in this study aligns strongly with existing literature, confirming the superior precision of USG for estimating spinal needle insertion depth. Our findings show a minimal mean bias of -0.0545 cm with narrow limits of agreement, which is consistent with several other studies. Jain et al. [10] had reported a mean difference of just 0.007 cm, while a systematic review by Perlas et al. [7] noted that most studies found mean differences of less than 3 mm. This figure contrasts sharply with the wider limits of agreement found for Stocker’s formula, a finding that supports the established limitations of this method in the literature. As highlighted by Nair et al. [6], a simple formula based on weight or BMI cannot provide the greater insight into individual anatomical variations, such as skin thickness and fat distribution, that USG offers. Our results, therefore, reinforce the consensus that USG is a more precise and reliable tool for this purpose.

By providing a preprocedural estimate of the required needle depth, accurate SSD measurement facilitates appropriate needle length selection, thereby minimizing the number of attempts needed to access the subarachnoid space. A study done by Dhanger et al. [11] shows that conventional landmark-based methods for estimating SSD have demonstrated limitations, with failure rates reported in the range of 20-37%, attributable to the significant variability in individual patient anatomy.

The study’s key findings highlight a strong relationship between USG measurements and the AD required for spinal needle insertion, demonstrating a high degree of accuracy with a correlation coefficient of 0.94 (p < 0.001). While Stocker’s formula also exhibited a strong correlation with actual needle depth, the association was slightly less robust, with a correlation coefficient of 0.86 (p < 0.001).

Utilizing an incorrect needle depth during spinal anesthesia procedures carries the risk of either suboptimal drug spread or a failed blockade. Conversely, precise SSD measurement ensures that local anesthetics are delivered effectively to the subarachnoid space, preventing the occurrence of partial or incomplete blocks.

USG measurements and AD of needle depth insertion measurements: skin-to-subarachnoid space distance

Our study demonstrates a strong correlation between USG-guided depth measurements and actual needle insertion depth during spinal anesthesia (r = 0.94, p < 0.001), confirming USG as a highly reliable method for estimating the SSD. The findings of this study align with a growing body of evidence supporting the use of USG-guided measurements in predicting spinal needle insertion depth with high accuracy. Previous studies by Sanguanwit et al. [12] and Girimurugan et al. [13] have demonstrated that preprocedural USG assessment significantly improves the precision of spinal anesthesia placement, reducing the number of failed attempts and complications.

A study by Devkota et al. [14] reported a strong correlation (r = 0.96, p < 0.001) between USG-measured depth and actual needle insertion depth, comparable to our study’s findings (r = 0.94, p < 0.001). Similarly, Gnaho et al. [15] found a significant correlation (r = 0.982) between space measured by USG and needle measurements. Furthermore, Bhatia et al. [16] had also documented the significant correlation (r = 0.953) between the USG depth and needle depth. Our study reinforces these conclusions, highlighting the high reliability of USG for individualized depth assessment.

Stocker’s formula measurements and AD of needle depth insertion measurements of skin-to-subarachnoid space distance

Stocker’s formula, which predicts SSD based on patient weight, has shown a reasonable correlation with actual needle insertion depth. In our study, the correlation coefficient was reported as r = 0.86, indicating a strong relationship between the predicted and ADs. However, while Stocker’s formula provides a useful approximation, it is slightly less precise than USG-guided measurements. For instance, Nair et al. [6] had shown that the mean SSD calculated using Stocker’s formula was 4.92 ± 0.6 cm, compared to 4.47 ± 0.6 cm measured by USG and 4.81 ± 0.6 cm for actual needle insertion depth.

Prior investigations have generally shown significant, though weaker, correlations between Stocker’s formula and observed needle depth. The study by Nair et al. [6] reported a correlation coefficient of 0.61, and another study by Girimurugan et al. [13] found a correlation coefficient of 0.736. These values, while indicative of a meaningful relationship, are lower than the correlation coefficient of 0.86 observed in our study. These variations in findings may be attributable to differences in population demographics, spinal anatomy, or the techniques used to measure SSD.

Compared to other anthropometric formulas, such as those developed by Craig, Abe, and Chong, Stocker’s formula demonstrates superior agreement with actual SSD, particularly within the Indian population by Girimurugan et al. [13] and the South Asian population by Khandelwal et al. [17]. Our study’s results are consistent with these findings, further supporting the Stocker’s formula clinical utility in preprocedural planning.

Stocker’s formula measurements and USG measurements of skin to subarachnoid space distance

While formulas like Stocker’s offer a convenient method for estimating the depth of needle insertion, the present study consistently demonstrates the enhanced precision of USG measurements. USG measurements with a correlation coefficient of 0.958 have been shown to outperform established formulas such as Stocker’s and Craig’s formulas, which have demonstrated weaker correlations (r = 0.736 and r = 0.656, respectively) in the study by Girimurugan et al. [13].

In our study, there is a strong positive correlation (r = 0.87), and the highly significant p-value (p < 0.001, Pearson correlation test) strongly suggests that there is a robust agreement between the depth of needle insertion as measured by USG and the depth estimated using Stocker’s formula. Whereas in a study by Nair et al. [6], comparing Stocker’s formula and USG measurements with a correlation coefficient value of 0.61, which shows a positive correlation with our study, yet a slightly lower correlation coefficient value than our study.

While our correlation analysis demonstrated superior performance of USG (r = 0.94) compared to Stocker’s formula (r = 0.86), the ANOVA revealed no statistically significant differences in mean depths between methods. This apparent contradiction can be explained by the strong agreement among all three methods in terms of absolute values, with mean differences of only 0.06 cm between USG and AD. The clinical relevance of this small difference may be limited for routine procedures but could be significant in challenging cases or when precise needle placement is critical.

Current research consistently demonstrates that the application of USG guidance for spinal procedures is particularly beneficial in patients with challenging anatomical landmarks, such as those with obesity. Khoo et al. [18] have shown that USG guidance dramatically improves first-attempt success rates in obese patients. In some cases, success rates have been shown to increase from as low as 10% with traditional palpation to over 42% with the use of USG [19]. These findings collectively affirm the value of USG as a tool to overcome the difficulties associated with landmark palpation in specific patient populations.

Regarding implementation considerations, USG-guided depth estimation requires initial equipment investment, operator training (20-30 supervised procedures), and additional procedural time (two to three minutes per case). However, these costs may be offset by reduced complications, fewer failed attempts, and improved patient satisfaction, particularly in high-risk populations.

Limitations

The study was restricted to adults aged 18-60 years with ASA Physical Status I or II, excluding those with more complex health conditions or spinal deformities, thus limiting the generalizability of the findings to broader patient populations. This study’s use of convenience sampling introduces a risk of selection bias, which may also limit the generalizability of our findings. The data was collected by a single anesthesiologist, ensuring internal consistency but raising concerns about external validity and inter-operator variability. Future research should establish standardized training protocols and assess inter-operator reliability to confirm the reproducibility of these findings across different clinical settings. Furthermore, the absence of data on long-term outcomes or complications limits insights into the procedure’s broader impact on patient safety and its potential to reduce complications.

## Conclusions

The findings of the study demonstrated that USG guidance, actual needle insertion depth, and Stocker’s formula yield equivalent mean estimates of SSD, with USG demonstrating the highest precision and strongest correlation to actual needle depth. While this suggests potential clinical utility, correlation does not imply causation, and direct evidence of improved safety outcomes, such as reduced complications or failed attempts, was not measured in this study. The clinical significance of the small mean difference (0.06 cm) requires further investigation in controlled trials. Obesity substantially increases insertion depth across all methods, reinforcing the value of USG in high-BMI patients. We recommend routine preprocedural USG localization to enhance accuracy, reduce procedural time and needle passes, and improve patient safety, particularly in obese or anatomically challenging populations. Future studies should explore automated USG algorithms and assess clinical outcomes associated with USG-guided depth prediction.
